# Current Patient Management for Bacterial Meningitis Simultaneously Affected by Stroke

**DOI:** 10.7759/cureus.69526

**Published:** 2024-09-16

**Authors:** Summer Wong, Allen Gee, Yasmine T Bazzi, Mariah Hamby, Jason Zheng, Harper Henderson, McKenna Schaar, Cyril Mina, Irene Kamel, Kevin Chun, Yuri Zagvazdin

**Affiliations:** 1 Department of Neurology, Nova Southeastern University Dr. Kiran C. Patel College of Osteopathic Medicine, Fort Lauderdale, USA; 2 Department of Medical Education, Nova Southeastern University Dr. Kiran C. Patel College of Allopathic Medicine, Fort Lauderdale, USA

**Keywords:** anticoagulant, bacterial meningitis, cerebrovascular complications, neurology, stroke

## Abstract

Bacterial meningitis (BM) is a critical central nervous system infection characterized by increased risks of complications and potentially fatal outcomes. The chances of full recovery are significantly reduced in the presence of concomitant neurovascular complications such as ischemic and hemorrhagic strokes, intracerebral hemorrhage, and cerebral sinus thrombosis. Effective treatment of BM requires a targeted approach that simultaneously addresses the causative pathogen and manages the neurologically related complications. However, clinicians continue to face challenges in determining optimal pharmacotherapy for these patients. The presence of neurological complications is pivotal in determining patient outcomes, contributing to high disability and mortality rates. Early medical management is crucial and begins with essential stabilization followed by rapid diagnostic testing and the administration of empirical broad-spectrum antimicrobial therapy. The use of targeted antibiotics based on culture results is standard, with adjunct therapies such as dexamethasone and, in some cases, anticoagulants playing supportive roles. Despite the deployment of such comprehensive therapeutic strategies, the variability in treatment response and the high incidence of adverse outcomes necessitate ongoing research. This includes exploring novel therapeutic approaches and enhancing current clinical practices through retrospective studies and clinical trials to mitigate the high morbidity and mortality rates associated with BM and its complications. Strategic management is crucial for patient recovery, considering the substantial risk, a broad spectrum of complications, and fatal outcomes from this meningeal infection and stroke. The use of antimicrobial therapy with intravenous adjunct dexamethasone is the current standard of care for patients with cerebrovascular complications and acute BM. In addition, other options that can provide benefits in complicated cases include anticoagulants and neurosurgery. Further investigation into treatment algorithms for patients with meningeal infection complicated by stroke and/or increased intracranial pressure is still needed.

## Introduction and background

Bacterial meningitis (BM) is a severe infection affecting the central nervous system, characterized by inflammation of the leptomeninges and tissues surrounding the brain and spinal cord [1]. The estimated annual incidence of BM ranges from 0.6 to four cases per 100,000 adults, with a higher occurrence observed among the elderly in developing countries [2]. This condition can lead to severe neurological damage, life-threatening complications, and sometimes fatal outcomes [3,4]. Causative agents include a range of gram-negative and gram-positive bacteria, each with varying predilections for different age groups. The most prevalent pathogens responsible for the condition’s morbidity and mortality include *Streptococcus pneumoniae *(36.5%), *Neisseria meningitidis* (28.8%), and *Streptococcus agalactiae* (15.4%) [5]. Other bacterial species, such as *Listeria monocytogenes* and *Group B Streptococcus*, can also cause meningitis, specifically in neonates and immunocompromised individuals [5].

Risk factors potentially impacting outcomes adversely in patients with BM encompass several elements: advanced age, a history of chronic diseases, including immunocompromised states, the presence of neurological or cerebrovascular complications, decreased consciousness at admission, the specific causative agent, and drug resistance [6, 7]. Notably, BM caused by *Streptococcus pneumoniae* is linked to a poorer prognosis, heightened neurological complications, and an elevated risk of cerebral infarction; 29.77% of mortality is caused by this germ [8-10]. 

Although BM is infrequent in adults, the associated mortality rate is as high as 37% and is therefore considered a medical emergency [11,12]. The presence of concurrent neurovascular accidents, such as hemorrhagic and ischemic stroke, significantly reduces the likelihood of recovery [11,13,14]. Neurological complications, including altered mental status, cerebral edema, increased intracranial pressure, hydrocephalus, seizure, hearing loss, focal neurological deficits, and intellectual impairment, have been reported in around 30% of patients with BM [2]. Despite the use of vaccinations, antibiotics, and corticosteroids that have been shown to reduce the prevalence of BM and its complications, up to 50% of patients still develop cerebrovascular injuries [3,7,9,12,13]. Clinicians often face challenges in accurately and promptly diagnosing BM due to its non-specific signs and symptoms. The classical triad of fever, headache, and nuchal rigidity, which is a hallmark of the condition, is present in only 44% of patients [2].

Therefore, the management of BM necessitates a comprehensive approach that addresses both the causative pathogen and potential neurological complications such as stroke, increased intracranial pressure, and seizures [15,16]. Furthermore, the risk of long-term sequelae highlights the importance of a follow-up and assessment of cognitive and executive functions [1]. Owing to the substantial morbidity and mortality associated with the natural course of these conditions, there is an acute interest within the medical community in identifying alternative therapeutic interventions. This paper aims to explore the etiology of BM complicated by stroke and the current therapeutic approaches in management within the clinical setting.

## Review

Complications of BM

Cerebral vasculopathy, such as ischemic and hemorrhagic strokes, intracerebral hemorrhage, and cerebral sinus thrombosis, frequently complicate BM and significantly contribute to high rates of disability and mortality in affected patients. Infarction to the brain, predominantly caused by *Streptococcus pneumoniae*, contributes significantly to adverse clinical outcomes and typically develops within the initial days of the disease [9,17]. Ischemic strokes represent the most prevalent cerebrovascular complication in BM patients, occurring in both early and late recovery stages. Cerebrovascular complications and ischemic strokes are present in up to 15% and 10% of BM cases, respectively [8]. In addition, a predilection exists for cerebral infarctions in patients with pneumococcal meningitis compared with other infectious meningitis [18]. Moreover, case reports have documented intracranial and subarachnoid hemorrhages, intracranial arterial stenosis, and cerebral venous thrombosis as potential complications that are not well understood yet often associated with a poor prognosis [8,17,19].

Medical management for BM

The primary approach to treating BM is medical management; initial steps include resuscitation and stabilizing the patient’s airway, breathing, and circulation. It is imperative to collect blood and cerebrospinal fluid (CSF) samples promptly and prior to the administration of any empirical antimicrobial therapy [2]. Targeted antibiotic treatment may be started once culture results are produced [20]. Prognostic evaluations frequently include assessments of CSF white blood cell (WBC) counts to anticipate ischemic complications [9,20]. Low levels of white blood cells (WBCs) in CSF have been demonstrated to be both a risk factor for unfavorable outcomes and a robust predictor of mortality in these patients [9].

To improve prognosis, a prompt diagnosis and timely administration of appropriate antimicrobial therapy is paramount [8]. Broad-spectrum antibiotics such as ceftriaxone, ampicillin, and vancomycin were deployed while waiting for blood and CSF culture and sensitivity results [10,12]. If *herpes simplex virus* (HSV) or *varicella-zoster virus* (VZV) is suspected as the causative organism, acyclovir may be included in the treatment regimen. This combination has been reported to reduce the probability of hearing loss and preserve neurological function [2]. In addition, the use of meropenem, linezolid, and narrow-spectrum antibiotics such as metronidazole, clindamycin, and high-dose penicillin (2.4 x 107 IU/day) for managing anaerobic bacteria and gram-positive microorganisms *Streptococcus constellatus*, *Streptococcus anginosus*, and *Streptococcus pneumoniae* in patients with BM complicated by stroke has been reported [10,21]. However, clinicians should exercise caution with oral penicillin, as it may exacerbate cerebrovascular processes such as aneurysm, vasculopathy, thrombosis, and subarachnoid hemorrhage [21].

Approximately one-third of patients with BM experience seizures, a consequence of bacterial toxins and the neurochemical alterations resulting from persistent inflammation, both of which contribute to a lowered seizure threshold [2]. Therefore, the addition of anti-epileptics such as levetiracetam may be employed for the management of seizures in patients with BM. There is insufficient data to support its use for seizure prophylaxis or preventing seizure recurrence during the recovery period; this decision is based on clinical judgment [22].

The practice of inducing hypothermia (32-34°C for 48 hours) in patients with severe BM was hypothesized to enhance clinical outcomes, as gauged by the Glasgow Coma Scale (GCS). However, this approach has been proven ineffective and subsequently discontinued due to concerns regarding patient safety [23].

Adjunctive therapy: dexamethasone

Adjunctive therapies such as dexamethasone have emerged as the primary pharmacotherapy to attenuate the inflammatory response caused by BM and have been administered with some success and variable efficacy [24]. The lack of randomized controlled trials and mixed outcomes have led to the case-by-case assessment of this treatment option [7,19,25-27]. Dexamethasone has been shown to reduce the risk of unfavorable outcomes and mortality in certain patient subgroups with BM, especially those with pneumococcal meningitis who presented earlier with the disease, preventing hearing loss and other neurological complications [28]. Based on the current evidence, it is widely accepted that dexamethasone should be administered to all adult patients with BM before or in combination with the first dose of antibiotics [24].

Despite its wide use, criticism has been directed at using dexamethasone in cases of antibiotic-resistant pneumococcal strains, as it could hinder CSF sterilization [27,29-31]. The lack of a standardized approach has also been cited as the main reason for caution regarding the protective effect of dexamethasone on the clinical outcome of BM [24,25]. Larger studies are needed to better understand the impact of symptom duration, disease severity, and the timing of antibiotic administration to the initiation of dexamethasone. 

Dexamethasone demonstrates potential in preventing and treating neurological complications in BM; however, its efficacy varies across patient demographics. For example, in developing countries, the impact of dexamethasone in treating BM is often diminished due to factors such as malnutrition, a delay in diagnosis, and chronic conditions such as *human immunodeficiency virus (*HIV) infection. Consequently, the role of dexamethasone as an adjunctive treatment to antibiotics in treating BM may be limited to developed countries [2].

Adjunctive therapy: anticoagulants

Ancillary anticoagulant therapy has been cautiously utilized to mitigate mortality risk associated with thromboembolic events [10]. For example, the use of recombinant human-activated protein C (drotrecogin alfa) provided substantial benefits in patients with BM by reducing mortality, stroke, infarctions, inflammation, and arterial stenosis from cerebral purulence [32]. However, the role of anticoagulants remains controversial due to the potential for intracranial hemorrhage, which can occur in 3% of adult patients with BM. In a study by Mook-Kanamori et al., there was a fivefold increase in developing intracranial hemorrhage with anticoagulant use at the time of admission. Patients with intracranial hemorrhage had a higher rate of mortality [63%] and unfavorable outcomes (96% vs. 34%) compared to patients without such complications [26]. The association of anticoagulants with an increased risk of hemorrhagic complications and higher mortality rates has been discussed in several other studies [10,33,34]. Contrarily, Deliran et al. observed no relationship between the use of anticoagulant therapy and intracerebral hemorrhage, warranting further investigation into the risks and benefits of anticoagulant therapy in these patients [19].

Role of imaging in BM

Imaging modalities such as computed tomography (CT) or magnetic resonance imaging (MRI) of the brain must be considered in patients who present with severely altered mental status, focal neurological deficits, seizures, evidence of increased intracranial pressure, or if the patient is immunocompromised [2,8,35]. An MRI is also the most sensitive tool for diagnosing rare cases of meningitis, such as community-acquired *E. coli *meningitis with complications such as ventriculitis and cerebral vasculitis [35]. In a subset of patients, magnetic resonance angiography (MRA) or computed tomography angiography (CTA) may be indicated to locate ischemic cerebrovascular complications [9]. Finally, detecting changes in blood flow at the base of the brain can be useful in patients with cerebral vasculopathy due to BM [9].

Role of surgery in BM

While medical management is the primary treatment for BM, incorporating neurosurgical consultations is crucial for patients presenting with surgically treatable lesions on brain CT scans concerning neurological symptoms and complications, or insufficient response to initial antibiotic therapy. Neurological complications warranting such consultation include brain abscess, elevated intracranial pressure, intracranial hemorrhage, hydrocephalus, and aneurysms.

In a case study by Lim et al. (2019), a patient with a BM infection displayed signs of stroke, increased intracranial pressure, and brain herniation due to global cerebral edema. This patient's prompt referral to neurosurgery was found to play a pivotal role in their treatment. The timely intervention of neurosurgery significantly reduced the patient's risk of further complications. As a result of this expedient and specialized care, the patient experienced a substantial recovery. By day 30, they were discharged with regained independence and the ability to perform activities of daily living (ADLs) effectively. This case underscores the importance of quick neurosurgical consultation in complex BM cases to improve patient outcomes [2].

Clinical and research implications 

Retrospective studies from Taiwan and Denmark have established a clinical link between stroke and meningitis. In Taiwan, patients with stroke had a 2.55 times higher incidence of meningitis than those without stroke [15]. Similarly, a retrospective population-based cohort study found that stroke patients had higher in-hospital mortality rates, unfavorable outcomes, and long-term complications than those without stroke. Most notably, there is no significant association between atherosclerosis risk factors and stroke in patients with community-acquired BM [7,8].

This review reflects the current knowledge of therapy, offering insights that can be considered when planning future clinical trials or treating patients with meningeal infections complicated by stroke. Currently, there is no universal agreement on the protocol for managing these complicated cases, which necessitates physicians to use their own discretion. In addition, there is some uncertainty about standards and options in therapy for patients with BM and its complications. While dexamethasone is the first-line anti-inflammatory treatment, there is still controversy regarding the efficacy of this drug in reducing mortalities and morbidities [3,13]. Given the statistically significantly higher mortality rates seen in complex BM cases, there is clearly a need for further work on developing optimal practices for these conditions.

Finally, exploring the use of clinical scales for standardizing evaluations in BM cases is essential, as this aspect has historically been underdeveloped. The GCS has traditionally been the sole assessment tool for clinical status in BM, but it fails to account for cerebrovascular complications frequently linked with worse outcomes. Utilizing scales like serial transcranial Doppler (TCD) recordings, the Scandinavian Stroke Scale (SSS), and the Hunt and Hess Scale (HH) in future clinical trials could standardize data collection, assess therapy responses, predict clinical outcomes, and evaluate both neurological and cerebrovascular complications [6,36]. In addition, the development of a risk prediction nomogram model has demonstrated clinical significance in early stroke detection among patients with BM [37].

## Conclusions

BM and stroke are medical emergencies that require prompt diagnosis and early intervention. This review synthesizes the existing literature on the epidemiology, etiology, and clinical outcomes of BM co-occurring with cerebrovascular accidents. Current guidelines are designed to treat both BM and stroke together, with a focus on addressing the underlying infection. Our review suggests that integrating intravenous corticosteroids, anticoagulants, and advanced neuroimaging techniques is a reasonable approach for providing additional therapeutic benefits in complicated cases resistant to standard treatments. Future randomized controlled trials and additional studies are necessary to gain more evidence for optimizing the recovery of patients diagnosed with BM complicated by stroke.
